# Independent Risk Factors for Sepsis-Associated Cardiac Arrest in Patients with Septic Shock

**DOI:** 10.3390/ijerph18094971

**Published:** 2021-05-07

**Authors:** Won Soek Yang, Youn-Jung Kim, Seung Mok Ryoo, Won Young Kim

**Affiliations:** 1 Department of Emergency Medicine, Hallym University Sacred Heart Hospital, Hallym University College of Medicine, Anyang 24252, Korea; wsyang@hallym.or.kr; 2 Asan Medical Center, Department of Emergency Medicine, University of Ulsan College of Medicine, Seoul 44610, Korea; yjkim.em@gmail.com (Y.-J.K.); chrisryoo@naver.com (S.M.R.)

**Keywords:** sepsis, septic shock, cardiac arrest, mortality

## Abstract

The clinical characteristics and laboratory values of patients with septic shock who experience in-hospital cardiac arrest (IHCA) have not been well studied. This study aimed to evaluate the prevalence of IHCA after admission into the emergency department and to identify the factors that increase the risk of IHCA in septic shock patients. This observational cohort study used a prospective registry of septic shock patients and was conducted at the emergency department of a university-affiliated hospital. The data of 887 adult (age ≥ 18 years) septic shock (defined using the Sepsis-3 criteria) patients who were treated with a protocol-driven resuscitation bundle therapy and were admitted to the intensive care unit between January 2010 and September 2018 were analyzed. The primary endpoint was the occurrence of sepsis-associated cardiac arrest. The patient mean age was 65 years, and 61.8% were men. Sepsis-associated cardiac arrest occurred in 25.3% of patients (*n* = 224). The 28-day survival rate after cardiac arrest was 6.7%. Multivariate logistic regression identified chronic pulmonary disease (odds ratio (OR) 2.06), hypertension (OR 0.48), unknown infection source (OR 1.82), a hepatobiliary infection source (OR 0.25), C-reactive protein (OR 1.03), and serum lactate level 6 h from shock (OR 1.34). Considering the high mortality rate of sepsis-associated cardiac arrest after cardiopulmonary resuscitation, appropriate monitoring is required in septic shock patients with major risk factors for IHCA.

## 1. Introduction

Sepsis is an infection-induced clinical syndrome. The global prevalence of sepsis is between 29.5 and 51% in multinational studies for intensive care unit patients [1,2].

In the United States, >200,000 patients experience in-hospital cardiac arrest (IHCA) yearly and only 20% of these patients are discharged alive [3,4,5,6]. In most cases (65%), cardiac arrest has a cardiac origin; however, between 13% and 27% of cases occur in adult patients with underlying sepsis [7,8,9].

Considering the cardinal pathophysiologic features of sepsis, cardiopulmonary resuscitation in patients with sepsis is challenging and usually unsuccessful, with a high risk of cerebral anoxic damage [7,8,10,11]. Recent registry data suggest that survival from sepsis-associated IHCA has improved during the last two decades; however, patients with sepsis continue to have worse outcomes than patients without sepsis [8,10]. To date, few studies have specifically evaluated the risk factors for the occurrence of sepsis-associated IHCA. Moreover, most of the studies on the epidemiology and outcomes of patients with sepsis who develop IHCA are limited by their use of data extrapolated from cardiac arrest registries, few of which specify sepsis as a subgroup of interest [4–6, 10–12]. In addition, these studies are limited by the variety of sepsis-related diagnoses and changes in the definition of sepsis [10,11,12,13,14,15,16]. The aim of this study was to evaluate the prevalence of IHCA in septic shock patients after admission into the emergency department and to determine the factors associated with an increased risk of IHCA in patients with septic shock, as defined by the Sepsis-3 criteria.

## 2. Methods

### 2.1. Study Design and Population

**Figure 1:**
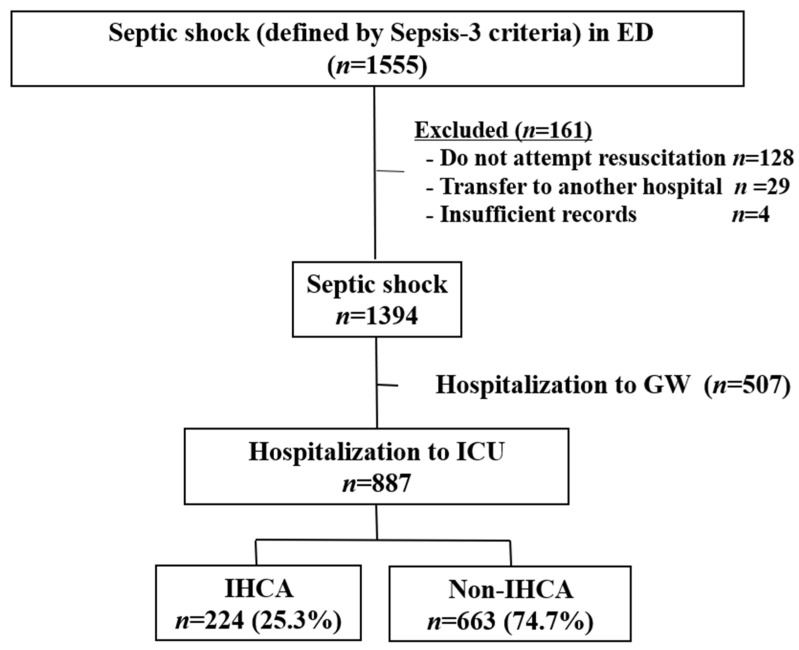
Flowchart of patient inclusion. Abbreviations: ED, emergency department; GW, general ward; ICU, intensive care unit; IHCA, in-hospital cardiac arrest.

This retrospective cohort study, based on a prospective registry of septic shock, was conducted at the emergency department of a university-affiliated hospital in South Korea with an annual patient volume of >110,000. This registry included all consecutive adult (age ≥ 18 years) patients with septic shock who were diagnosed in the emergency department of Asan Medical Center since January 2010. The registry contains demographic findings and clinical features, including laboratory results, details of treatment, and prognosis such as mortality. Septic shock was defined according to the Sepsis-3 criteria as follows: refractory hypotension (systolic blood pressure < 90 mmHg, mean arterial pressure < 65 mmHg, or systolic blood pressure decrease > 40 mmHg) and hypoperfusion (serum lactate level ≥ 4 mmol/L) after adequate intravenous fluid challenges (20–30 mL/kg). Patients who were admitted to the intensive care unit between January 2010 and September 2018 were included and analyzed for the occurrence of cardiac arrest during hospitalization. All patients were treated for septic shock with a protocol-driven resuscitation bundle therapy, including crystalloid administration, blood culture, broad-spectrum antibiotics, vasopressors, lung-protective ventilation, glucocorticoids, and surgical intervention (if indicated). Patients transferred to another hospital during treatment and those who refused resuscitation treatment were excluded (Figure 1).

This study was approved by the research ethics committee of the hospital, which waived the requirement for informed consent because of the retrospective design.

### 2.2. Data Collection

Demographic and clinical data, including age, sex, comorbidities, vital signs at septic shock diagnosis, source of infection, laboratory test findings, initial serum lactate level, and Sequential Organ Failure Assessment (SOFA) scores, were collected. Initial serum lactate levels were measured at shock recognition, and a follow-up measurement was performed 6 h after the initial measurement. The primary endpoint was the occurrence of sepsis-associated cardiac arrest, which was defined as the first event of pulseless cardiac activity of any cardiac rhythm occurring in septic shock patients during intensive care unit admission. If patients or family members agreed to DNR (do not resuscitate) or POLST (physician orders for life-sustaining treatment), they were excluded from the study. Patients admitted to the intensive care unit were transported to the general ward upon the improvement of the clinical symptoms of septic shock, as judged by the clinicians.

### 2.3. Statistical Analysis

The normality of data distributions was evaluated using the Kolmogorov–Smirnov test to select the appropriate parametric and nonparametric statistical methods. Categorical variables were analyzed using the chi-square test or Fisher’s exact test. Continuous variables were expressed as medians (25th–75th percentile) and were analyzed using the Mann–Whitney U test. We used age, sex, all variables in baseline and clinical characteristics, and laboratory findings for univariate logistic regression analysis of risk factors for the occurrence of IHCA. Considering the problem of collinearity, we analyzed each variable separately without using a SOFA score. Independent risk factors associated with IHCA were evaluated using multivariate backward stepwise logistic regression after adjustment for confounding factors, defined as factors found to be significant in univariate analysis based on a type I error of 0.1. We presented the odds ratios (ORs) with 95% confidence intervals (CIs) for each model. The SPSS statistical software package for Windows (version 26.0; SPSS Inc., Chicago, IL, USA) was used for all analyses.

## 3. Results

Of the 1555 patients with septic shock during the study period, 128 patients with a DNR order, 29 patients who were transferred to another hospital, and 4 patients with insufficient records were excluded. The remaining 1394 patients were enrolled. The rest of the 887 patients with septic shock who were admitted to the intensive care unit were finally included. The incidence of IHCA in our patients was 25.3% (*n* = 224) (Figure 1). The median time from emergency department visit to the occurrence of cardiac arrest was 4 (2–12) days.

### 3.1. Clinical Characteristics

The baseline and clinical characteristics of septic shock patients who developed IHCA and those who did not are summarized in Table 1. The median age of the patients was 65 years, and 61.8% of the patients were men. Among the comorbidities, chronic pulmonary disease was more frequent in the IHCA group (12.9% vs. 8.6%, *p* = 0.015). Among the vital signs of shock recognition, respiration rate was higher in the IHCA group (22 (20–28) breaths/min) than in the non-IHCA group (20 (20–26) breaths/min) (*p* = 0.031). The hepatobiliary tract and urinary tract were more frequently identified as sources of infection in the non-IHCA group (25.8% vs. 12.5%, p < 0.001; 11.9% vs. 5.4%, *p* = 0.005). An unknown infection source was more frequent in the IHCA group (15.2% vs. 5.8%, *p* < 0.001), and the SOFA score at day one was significantly higher in the IHCA group (10 (8–13) vs. 9 (6–11), p < 0.001).

Table 2 compares the laboratory findings between septic shock patients with and without IHCA. Patients with IHCA had significantly higher serum levels of creatinine, aspartate aminotransferase, C-reactive protein, lactate at shock recognition, and lactate at 6 h than patients without IHCA (Table 2).

Table 3 compares the treatment and clinical outcomes between septic shock patients with and without IHCA. A significantly higher use of steroid and renal replacement therapy was observed in IHCA patients. The duration of mechanical ventilation was longer in patients who developed IHCA. Moreover, the rate of mortality at 28 days was significantly higher in the IHCA group (93.3%) than in the non-IHCA group (5%) (*p* < 0.001). The 90-day mortality in the IHCA group was also higher than that in the non-IHCA group (98.7% vs. 50.0%, *p* < 0.001).

### 3.2. Associated Risk Factors for the Development of IHCA

The results of univariate and multivariate logistic regression analyses performed to identify the risk factors for the development of IHCA in patients with septic shock are presented in Table 4. Age, sex, and all variables in Table 1 and 2 were analyzed for univariate logistic regression analysis of risk factors for the occurrence of IHCA. All variables with *p*-values less than 0.1 are presented in Table 4; the rest were omitted.

Variables with *p* < 0.1 in the univariate analysis were adjusted in the multivariable analysis. In multivariate logistic regression analysis, the following factors were found to be independently associated with the development of IHCA: preexisting hypertension (OR 0.48, 95% CI 0.30–0.80), preexisting chronic pulmonary disease (OR 2.01, 95% CI 1.19–3.58), hepatobiliary infection source (OR 0.25, 95% CI 0.11–0.54), unknown infection source (OR 1.82, 95% CI 1.01–3.47), serum level of C-reactive protein (OR 1.03, 95% CI 1.01–1.04), and serum level of lactate 6 h from shock recognition (OR 1.34, 95% CI 1.22–1.47).

## 4. Discussion

Although coronary disease is the main etiology of cardiac arrest, approximately 13–27% of patients experience cardiac arrest related to infection such as sepsis, which has an even worse prognosis [5,8]. In this study, we observed the occurrence of IHCA in patients with septic shock diagnosed in the emergency department and admitted to the intensive care unit. We compared the clinical characteristics of the IHCA and non-IHCA groups. Characteristics such as chronic pulmonary disease, an unknown infection source, and serum lactate level 6 h from shock recognition had high odds for the occurrence of sepsis-associated IHCA, whereas a history of hypertension and a hepatobiliary infection source were associated with low odds for the development of sepsis-associated IHCA. These findings have potential clinical implications, such as in the early identification of high-risk patients with septic shock who would require intensive monitoring or aggressive management to prevent IHCA in the intensive care unit.

The 28-day mortality rate of septic shock patients admitted to the intensive care unit was 27.3% in this study. This is consistent with the mortality rate in the ANZICS cohort (22.0%) and that in other studies [17,18,19]. Although most of the previous studies on septic shock selected mortality as a primary outcome, the occurrence of IHCA does not have the same meaning as mortality. One early study included 73 patients with sepsis-associated IHCA, of whom 45% were initially resuscitated but only one patient survived to discharge compared with 7.8% of patients without sepsis who survived to discharge [16]. However, more recent data have shown that the survival rate from sepsis-associated IHCA has improved from 7.6% to 9.3% [10,11]. This is consistent with the present result that 6.7% of patients with sepsis-associated IHCA survived up to 28 days. Our study has several strengths as compared with previous studies. First, considering that the most common cause of death from septic shock is the withdrawal of treatment [20], our results can reflect the natural course of septic shock because withdrawing life support treatment from resuscitated patients is legally prohibited in South Korea. In addition, to our knowledge, almost no data exist on the occurrence of sepsis-associated IHCA from septic shock registries using the Sepsis-3 definition. Previous epidemiological data on sepsis-associated IHCA were derived from cardiac arrest registries that report only the proportion of cardiac arrest cases among patients with sepsis or related disease categories.

In previous studies, serum lactate level was related to mortality in septic shock patients [21,22,23,24]. In our study, the initial serum lactate level was a statistically significant factor in the occurrence of IHCA in univariate analysis but did not reach statistical significance in multivariate analysis. An increased serum lactate level indicates that the patient did not recover from shock despite receiving the best treatment in the intensive care unit and, thus, has the possibility of developing IHCA. Few previous studies performed a serial follow-up of serum lactate level to predict mortality in septic shock patients [18,25]. Serial testing of serum lactate level may be necessary to check the response to treatment and to predict the occurrence of unexpected IHCA.

Previous studies reported the correlation between serum C-reactive protein level and mortality of sepsis patients [26,27,28,29,30]. This is consistent with the present results, although we strictly selected the septic shock patients who met Sepsis-3 and were hospitalized in the intensive care unit.

In accordance with previous studies, patients with an unknown source of sepsis or a bacteremia infection had higher mortality rates than patients with known infection sources [31,32,33,34]. It is presumed that the difficulty in selecting the appropriate antibiotics and in the application of proper procedures and surgical intervention may have affected the treatment and prognosis.

This study had some notable limitations. Owing to the retrospective design, our study is limited by factors inherent in the collection, analysis, and interpretation of retrospective data despite the use of a prospective septic shock registry. In addition, although our cohort comprised a considerable number of strictly selected septic shock patients admitted to the intensive care unit from the emergency department, this study was conducted at a single institution, which limits the generalization of our results to other institutions or to the general population.

## 5. Conclusions

Cardiac arrest occurred after hospitalization in a quarter of septic shock patients in this study. Considering the high rates of mortality from sepsis-associated cardiac arrest after cardiopulmonary resuscitation, appropriate monitoring is required in patients with septic shock with major risk factors for cardiac arrest.

## Figures and Tables

**Table 1:** Comparison of baseline and clinical characteristics between the IHCA and non-IHCA groups.

Variables		Total Patients (*n* = 887)	Non-IHCA Group (*n* = 663)	IHCA Group (*n* = 224)	*p*-Value ^c^
Age, years		65.0 ± 12.6	65.2 ± 12.5	64.6 ± 12.8	0.951
Sex, male		548 (61.8)	410 (62.0)	138 (61.3)	0.558
Comorbidities					
Hypertension		307 (34.6)	241 (36.4)	66 (29.3)	0.055
Diabetes		245 (27.6)	191 (28.9)	54 (24.0)	0.163
Coronary disease		86 (9.7)	57 (8.6)	29 (12.9)	0.083
Chronic pulmonary disease		144 (16.3)	95 (14.4)	49 (21.8)	**0.015**
Liver cirrhosis		138 (15.6)	104 (15.7)	34 (15.1)	0.856
Chronic renal failure		66 (7.4)	49 (7.4)	17 (7.6)	0.922
Stroke		49 (5.5)	40 (6.0)	9 (4.0)	0.254
Active Malignancy ^a^		384 (43.3)	293 (44.2)	91 (40.6)	0.313
Vital signs at shock recognition					
MAP, mmHg	60 (53–66)		60 (53–66)	59 (51–67)	0.526
Pulse rate, breaths/min	111 (93–126)		110 (93–126)	112 (94–126)	0.888
Body temperature, °C	37.5 (36.6–38.3)		37.6 (36.7–38.4)	37.1 (36.4–38.1)	0.136
GCS	15 (12–15)		15 (15–15)	15 (3–15)	**0.050**
Source of sepsis					
Lung		286 (32.2)	203 (30.6)	83 (37.1)	0.083
Gastrointestinal tract		132 (14.9)	99 (14.9)	33 (14.7)	0.942
Hepatobiliary tract		199 (22.4)	171 (25.8)	28 (12.5)	**<0.001**
Urinary tract		91 (10.3)	79 (11.9)	12 (5.4)	**0.0** **05**
Bloodstream		50 (5.6)	32 (4.8)	18 (8.0)	0.092
Soft-tissue infection		39 (4.4)	30 (4.5)	9 (4.0)	0.852
Unknown		73 (8.2)	39 (5.8)	34 (15.2)	**<0.001**
Others ^b^		17 (1.9)	10 (1.5)	7 (3.1)	0.156
Severity					
SOFA score (day 1)		9 (7–12)	9 (6–11)	10 (8–13)	**<0.001**

^a^ The Haemostasis and Malignancy Scientific and Standardization Committee defines “active cancer” as cancer diagnosed within the previous six months; recurrent, regionally advanced, or metastatic cancer; cancer for which treatment had been administered within six months; or hematological cancer that is not in complete remission. ^b^ Other sources of infection included the central nervous system, catheter, and infectious endocarditis. ^c^ The *p*-value of this table was calculated from chi-square test or Fisher’s exact test for categorical variables and student T-test or Mann–Whitney U test for continuous variables. Values are expressed as mean ± standard deviation, median (interquartile range), or number (%). Statistically significant p-values are indicated in bold. Abbreviations: MAP, mean arterial pressure; GCS, Glasgow coma scale; SOFA, Sequential Organ Failure Assessment.

**Table 2:** Comparison of laboratory findings between the IHCA and non-IHCA groups.

Variables	Total Patients (*n* = 887)	Non-IHCA Group (*n* = 663)	IHCA Group (*n* = 224)	*p*-Value ^a^
White blood cell, ×10^3^/L	10.6 (4.3–19.2)	11.3 (5.3–20.0)	8.15 (2.3–17.4)	0.191
Hemoglobin, g/dL	11.2 (9.4–13.0)	11.3 ± 2.8	11.1 ± 2.8	0.196
Platelets, ×10^3^/L	140 (70–213)	147 (78–222)	120 (50–138)	**0.015**
Sodium, mmol/L	135 (131–138)	135 (131–138)	135 (130–138)	0.930
Potassium, mmol/L	4.2 (3.7–4.8)	4.1 (3.7–4.7)	4.4 (3.8–5.0)	0.170
Chloride, mmol/L	99 (95–103)	99 (95–103)	99 (94–103)	0.578
Creatinine, mg/dL	1.6 (1.1–2.7)	1.5 (1.0–2.6)	1.9 (1.3–3.0)	**0.032**
AST, U/L	47 (29–97)	46 (29–92)	49 (28–133)	**0.018**
ALT, U/L	30 (17–62)	30 (18–62)	28 (17–62)	0.096
Albumin, g/dL	2.6 (2.2–3.1)	2.7 (2.2–3.2)	2.8 (1.9–2.8)	0.423
Total bilirubin, mg/dL	1.2 (0.7–2.5)	1.3 (0.8–2.5)	1.2 (0.7–2.5)	0.128
Prothrombin time, INR	1.31 (1.15–1.58)	1.29 (1.14–1.53)	1.41 (1.21–1.77)	0.982
CRP, mg/dL	13.2 (4.9–23.17)	11.8 (4.2–21.6)	16.7 (7.1–25.7)	**<0.001**
Procalcitonin, mmol/L	15.8 (3.1–44.0)	15.8 (3.2–45.6)	15.8 (2.7–38.3)	0.341
D-dimer, mcg/mL	5.3 (2.7–12.0)	5.1 (2.5–11.6)	6.5 (3.2–13.9)	0.810
Troponin-I, ng/mL	0.05 (0.02–0.16)	0.05 (0.01–0.15)	0.05 (0.02–0.18)	0.613
pH	7.42 (7.34–7.47)	7.43(7.35–7.47)	7.38(7.24–7.46)	0.327
PCO_2_, mmHg	27 (22–33)	27 (22–32)	28 (22–33)	0.422
PO_2_, mmHg	75 (60–92)	75 (62–92)	72 (57–92)	0.922
Bicarbonate, mmol/L	18 (14–22)	19 (15–22)	17 (12–21)	**<0.003**
Initial lactate, mmol/L	4.6 (3.1–7.2)	4.4 (3.0–6.3)	6.1 (3.6–9.7)	**<0.001**
Lactate after 6 h, mmol/L	3.9 (2.4–5.9)	3.4 (2.2–5.0)	5.7 (3.1–9.2)	**<0.001**
Lactate normalization in 6 h	152 (17.1)	128 (19.3)	24 (10.7)	**<0.001**

^a^ The *p*-value of this table was calculated from chi-square test or Fisher’s exact test for categorical variables and student T-test or Mann–Whitney U test for continuous variables. Values are expressed as mean ± standard deviation, median (interquartile range), or number (%). : Statistically significant *p*-values are indicated in bold. Abbreviations: AST, aspartate aminotransferase; ALT, alanine transaminase; INR, international normalized ratio; CRP, C-reactive protein; PO_2_, partial pressure of oxygen; PCO_2_, partial pressure of carbon dioxide.

**Table 3:** Comparison of treatment and clinical outcomes between the IHCA and non-IHCA groups.

Variables	Total Patients (*n* = 887)	Non-IHCA Group (*n* = 663)	IHCA Group (*n* = 224)	*p*-Value ^a^
Steroid treatment	308 (34.7)	195 (29.4)	113 (50.4)	**<0.001**
RRT	313 (35.3)	174 (26.2)	139 (62.1)	**<0.001**
Duration of mechanical ventilation, days	2 (0–8)	0 (0–7)	3 (2–12)	**0.010**
Duration of ICU stay, days	5 (3–10)	5 (3–10)	4 (2–12)	0.721
Mortality at 28 days	242 (27.3)	33 (5.0)	209 (93.3)	**<0.001**
Mortality at 90 days	532 (60.0)	331 (50.0)	221 (98.7)	**<0.001**

^a^ The *p*-value of this table was calculated from chi-square test or Fisher’s exact test for categorical variables and student T-test or Mann–Whitney U test for continuous variables. Values are expressed as mean ± standard deviation, median (interquartile range), or number (%). : Statistically significant *p*-values are indicated in bold. Abbreviations: RRT, renal replacement therapy; ICU, intensive care unit.

**Table 4:** Logistic regression analysis of risk factors associated with IHCA in septic shock patients.

Variable	Univariate Analysis			Multivariate Analysis		
	OR	95% CI	*p*-Value ^a^	OR	95% CI	*p*-Value ^b^
Age	0.99	0.725–1.352	0.951			
Sex	0.996	0.985–1.008	0.558			
Hypertension	0.731	0.527–1.015	0.062	0.484	0.295–0.796	**0.0** **04**
Chronic pulmonary disease	1.674	1.140–2.458	0.009	2.058	1.185–3.575	**0.01** **0**
GCS	0.953	0.092–1.007	0.085			
Pulmonary infection source	1.426	1.039–1.957	0.028			
Hepatobiliary infection source	0.394	0.254–0.611	<0.001	0.246	0.113–0.536	**<0.001**
Urinary tract infection source	0.389	0.203–0.746	0.004			
Unknown infection source	2.842	1.734–4.656	<0.001	1.820	1.012–3.472	**0.0** **43**
Platelet count	0.998	0.997–1.000	0.015			
Creatinine	1.080	1.003–1.163	0.042			
AST	1.001	1.000–1.001	0.005			
CRP	1.027	1.014–1.040	<0.001	1.028	1.005–1.042	**0.0** **10**
Bicarbonate	0.946	0.920–0.972	<0.001			
Initial lactate	1.173	1.122–1.227	<0.001			
Lactate after 6 h	1.288	1.219–1.360	<0.001	1.338	1.221–1.465	**<0.001**

^a^ The *p*-Value from univariate logistic analysis of risk factors for IHCA. ^b^ The *p*-Value from multivariate logistic analysis of risk factors for IHCA. : Statistically significant *p*-values are indicated in bold. Abbreviations: GCS, Glasgow coma scale; AST, aspartate aminotransferase; CRP, C-reactive protein.

## Data Availability

Not applicable.
